# A dataset comprising four micro-computed tomography scans of freshly fixed and museum earthworm specimens

**DOI:** 10.1186/2047-217X-3-6

**Published:** 2014-05-16

**Authors:** Jennifer Lenihan, Sebastian Kvist, Rosa Fernández, Gonzalo Giribet, Alexander Ziegler

**Affiliations:** 1 Museum of Comparative Zoology, Department of Organismic and Evolutionary Biology, Harvard University, 26 Oxford Street, Cambridge, MA 02138, USA; 2 Ziegler Biosolutions, Fahrgasse 5, 79761 Waldshut-Tiengen, Germany

**Keywords:** MicroCT, Morphology, Anatomy, Taxonomy, Lumbricidae, *Aporrectodea*, μCT, Repository, Imaging

## Abstract

**Background:**

Although molecular tools are increasingly employed to decipher invertebrate systematics, earthworm (Annelida: Clitellata: ‘Oligochaeta’) taxonomy is still largely based on conventional dissection, resulting in data that are mostly unsuitable for dissemination through online databases. In order to evaluate if micro-computed tomography (μCT) in combination with soft tissue staining techniques could be used to expand the existing set of tools available for studying internal and external structures of earthworms, μCT scans of freshly fixed and museum specimens were gathered.

**Findings:**

Scout images revealed full penetration of tissues by the staining agent. The attained isotropic voxel resolutions permit identification of internal and external structures conventionally used in earthworm taxonomy. The μCT projection and reconstruction images have been deposited in the online data repository *GigaDB* and are publicly available for download.

**Conclusions:**

The dataset presented here shows that earthworms constitute suitable candidates for μCT scanning in combination with soft tissue staining. Not only are the data comparable to results derived from traditional dissection techniques, but due to their digital nature the data also permit computer-based interactive exploration of earthworm morphology and anatomy. The approach pursued here can be applied to freshly fixed as well as museum specimens, which is of particular importance when considering the use of rare or valuable material. Finally, a number of aspects related to the deposition of digital morphological data are briefly discussed.

## Data description

### Purpose of data acquisition

The present dataset constitutes the first attempt at comparative micro-computed tomography (μCT) scanning of earthworm (Annelida: Clitellata: ‘Oligochaeta’) specimens. When used in combination with staining techniques that permit enhancing soft tissue contrast [1], μCT could become a promising technique for resolving pervasive issues in earthworm taxonomy and systematics. To this end, the application of μCT to freshly fixed and museum specimens was evaluated, and results were compared with data derived from traditional dissection techniques. The main methodological and taxonomical results of the study are presented in an accompanying publication [2].

The aim of the present report is to provide the earthworm research community with a reference dataset for future analyses of soft-bodied organisms based on non-destructive imaging techniques. In addition, uninhibited data access and enforced data deposition, as practiced here, are briefly discussed.

### Scanned specimens

Scans of four lumbricid (‘Oligochaeta’: Lumbricidae) earthworm specimens are part of the present dataset. One freshly fixed and one museum specimen (stored in ethanol for several decades) were scanned for each of the two different species employed in the study, *i.e. Aporrectodea caliginosa* (Savigny, 1826) and *Aporrectodea trapezoides* (Dugès, 1828). All four specimens were stained using an ethanol-based phosphotungstic acid (PTA) solution, which was adapted from protocols described previously [3]. In order to increase the isotropic voxel resolution of the three-dimensional (3D) image stack, only the first ca. 35 segments of each specimen were scanned. These segments harbor all internal and external structures commonly used in earthworm taxonomy. Specific specimen data and supplementary image files have been deposited in the publicly accessible database of the Museum of Comparative Zoology, MCZbase (http://mczbase.mcz.harvard.edu/). In addition, hyperlinks to each specimen entry in MCZbase are provided on the dataset website in the *GigaScience Database* (*GigaDB*) online repository [4].

### Data acquisition and processing

The four scans were produced using a μCT system equipped with a cone-beam tungsten X-ray source (SkyScan 1173, Bruker microCT, Kontich, Belgium). The specific scanning parameters are provided in the accompanying publication [2], and can also be found in the log file (.log) of each dataset folder available for download at *GigaDB*[4].

Each scan resulted in a set of 960 projection images in tagged image file format (TIFF, .tif). No binning protocols were employed during data acquisition. The projection images covered 2240 × 2240 pixels at 16-bit dynamic range. Reconstruction of the two-dimensional (2D) projection images into a 3D volumetric image stack was performed using the software NRecon 1.6.6.0 (Bruker microCT, Kontich, Belgium). This program runs under the reconstruction engine NReconServer 1.6.6, which employs a Feldkamp algorithm for volumetric reconstruction [5]. The two reconstruction parameters with significant effect on the quality of the final data were ring artifact and beam hardening correction. The output format for the 3D volumetric image stacks was bitmap image file (BMP, .bmp) at 8-bit dynamic range and 2240 × 2240 pixel size. In order to reduce final file size, the volume of interest (VOI) function, a 3D cropping tool, was used to remove all uninformative parts of the data following reconstruction. This resulted in changes to the pixel dimensions of each reconstructed image stack, but did not lead to spatial distortions in any of the three dimensions. Further information on the contents and size of both the projection and the reconstruction data folders is provided in Table 1.

**Table 1:** **Overview of the earthworm dataset deposited in *GigaDB***

**Specimen**	**Isotropic voxel resolution**	**Projection folder files**	**Projection folder size**	**Reconstruction folder files**	**Reconstruction folder size**
*Aporrectodea trapezoides*	8.17 μm	960 × .tif	Uncompressed: 8.98 GB	2196 × .bmp	Uncompressed: 1.61 GB
MCZ IZ 24804					
Freshly fixed specimen		1 × .log	Compressed: 7.19 GB	1 × .log	Compressed: 0.66 GB
		1 × .tif		1 × .bmp	
		17 × .crv			
*Aporrectodea caliginosa*	9.95 μm	960 × .tif	Uncompressed: 8.98 GB	2194 × .bmp	Uncompressed: 1.18 GB
MCZ IZ 24805					
Freshly fixed specimen		1 × .log	Compressed: 8.03 GB	1 × .log	Compressed: 0.37 GB
		1 × .tif		1 × .bmp	
		33 × .crv		1 × .roi	
*Aporrectodea caliginosa*	13.15 μm	960 × .tif	Uncompressed: 8.98 GB	2226 × .bmp	Uncompressed: 0.70 GB
MCZ IZ 95557					
Museum specimen		1 × .log	Compressed: 7.67 GB	1 × .log	Compressed: 0.21 GB
		1 × .tif		1 × .bmp	
		14 × .crv		1 × .crv	
*Aporrectodea trapezoides*	8.17 μm	960 × .tif	Uncompressed: 8.98 GB	2226 × .bmp	Uncompressed: 2.87 GB
MCZ IZ 95901		1 × .log	Compressed: 8.37 GB	1 × .log	Compressed: 0.60 GB
Museum specimen		1 × .tif		1 × .bmp	
		9 × .crv		1 × .crv	

Explanation of the file types: .bmp = reconstructed images (multiple files), reference reconstruction (single file); .crv = preview file when setting projection or reconstruction parameters; .log = log file listing scan parameters; .roi = 2D region of interest (ROI) used to create a 3D volume of interest; .tif = projection images (multiple files), reference projection (single file).

### Data quality

The quality of the data was ascertained through visual inspection of the scout projection and reconstruction images. Primary criteria were i) the full penetration of the sample by the staining agent and ii) the absence of artifacts. Although a total of eight scans were obtained in the course of the study, four of these scans were either trial scans or showed significant artifacts [2]. Therefore, only the four most representative scans have been deposited in *GigaDB*. Nonetheless, these scans represent the full taxonomic and morphological breadth of species and sample types included in the study. The imagery allows for an identification of numerous internal and external structures. No significant difference in the approach was observed when employing freshly fixed or museum specimens, nor between the two species analyzed.

### Potential uses

The potential uses of the dataset presented here include morphometric or volumetric analyses of internal organs, studies of ingested sediment particles, the possibility of online collaborative dataset annotation, or interactive data exploration using digital 2D and 3D visualization tools.

The methodological approach itself is suitable for high-throughput scanning of hundreds or even thousands of earthworm specimens as well as other soft-bodied organisms [2]. This would result in large morphological taxon sampling, one of the prerequisites for broad taxonomic and systematic studies. Furthermore, non-invasive imaging techniques such as μCT leave specimens intact and generate digital data suitable for online dissemination, an important condition for effective data mining.

## Availability and requirements

### Data availability

The dataset is available at *GigaDB* and has a citable digital object identifier (DOI) [4]. Each of the eight folders has been packed using tape archiver (tar, .tar), before being compressed using GNU zip (gzip, .gz). The folders can be individually downloaded using a set of tools, e.g. File Transfer Protocol (FTP).

**Dataset name:** MicroCT scans of freshly fixed and museum earthworm specimens

**Operating system:** Platform-independent

**License:** Creative Commons 0 (CC0) public domain dedication (https://creativecommons.org/publicdomain/zero/1.0/)

### Data requirements

Following download, the reconstructed images can, for example, be rapidly visualized using the ‘File:Import:Image Sequence’ command chain in the Java-based imaging software ImageJ (http://imagej.nih.gov/ij/). In addition, numerous other 2D and 3D visualization tools are available for free [6]. Given the size of the reconstructed image folders, a computer system with about 4 GB main random access memory (RAM) and 1 GB video RAM should be used.

## Discussion

The dataset presented here permits full open access both to μCT-derived raw data (here: the projection images) as well as derivative data (here: the reconstructed image stacks). The availability of μCT raw data files has been deemed important, primarily due to the rapid increase in the performance of reconstruction algorithms, which in the future could lead to improved data reconstruction [7]. Furthermore, one reviewer as well as the editor of the accompanying publication [2] requested data deposition for purposes of data transparency, which was achieved here through storage and archiving of the dataset in *GigaDB*[4]. Despite these advances, a lack of coherent policy for data archiving and enforced data deposition in digital morphology remains [8], and metadata standards for data gathered using non-invasive imaging techniques are still not available [7].

## Availability of supporting data

The dataset supporting the results of this article is available in the *GigaScience Database* online repository [4].

## Abbreviations

2D: Two-dimensional; 3D: Three-dimensional; BMP: Bitmap image file; CC0: Creative Commons 0 1.0 public domain dedication; DOI: Digital object identifier; FTP: File Transfer Protocol; GigaDB: GigaScience Database; GNU: GNU’s not unix; gzip: GNU zip; MCZ IZ: Museum of Comparative Zoology Invertebrate Zoology; μCT: Micro-computed tomography; PTA: Phosphotungstic acid; RAM: Random access memory; ROI: region of interest; TIFF: Tagged image file format; VOI: Volume of interest.

## Competing interests

The authors declare that they have no competing interests.

## Authors’ contributions

Conceived and designed the experiments: JL SK RF AZ. Performed the experiments: JL SK RF. Analyzed the data: JL SK RF AZ. Contributed reagents/materials/analysis tools: JL SK RF GG AZ. Wrote the paper: JL SK RF GG AZ. All authors read and approved the final manuscript.
